# Advanced glycation end products (AGEs) involvements in the development of frank Alzheimer’s disease (FAD)

**DOI:** 10.1002/alz.095169

**Published:** 2025-01-09

**Authors:** Vincent Oghenekevbe, O Olughor

**Affiliations:** ^1^ Faculty of Pharmacy, Department of Pharmaceutics and Industrial Pharmacy, University of Ibadan, Ibadan Nigeria

## Abstract

**Background:**

AGEs is one of the Maillard’s reaction products (MRPs) found in over‐processed common diets, causing food‐nutrients abnormal modifications. Some MRPs are potentially toxic or carcinogenic. MRPs present in stages: initial, intermediate and final. The aim of this presentation is to state roles played by AGEs in the development of AD as recent findings.

**Method:**

Through threaded literature review

**Result:**

1) MRPs and disease causing associations were first recognized by Swedish scientists in 2002 and involvement of AGEs in development of AD in 2010; 2) MRPs include: N'fructoselysine (furosine), 5‐ Hydroxymethyl furfural (HMF), acrylamide, heterocyclic amines and melanoidins; 3) some neurotransmitters are modified amino acids, known as monoamines; 4) some other neurotransmitters are unmodified amino acids; 5) certain short chain amino acids (peptides) function as neuromodulators, by being able to alter a neuron’s response to a neurotransmitter, or block its release; 6) a neurotransmitter’s effect depends upon its concentration, the types and numbers of receptors and ion channels on the receiving cells' membranes; 7) neurotransmitters affect each other’s levels, the same neurotransmitter can even have opposite effects on different types of cells; 8) AD is due to depleted Acetylcholine (Ach) neurotransmitter in the brain, causing imbalance. Ach coordinates functions in the brain by enhancing nerve transmission in a horizontal direction and also inhibiting transmission in a vertical direction; 9) Ach can both inhibit and stimulate because it can bind two types of receptors: on basket cells (to decrease levels of GABA production and stimulate horizontal nerve transmission) and on bipolar cells (to increase GABA production and strengthen the inhibition of vertical transmission).

**Conclusion:**

How AGEs disrupts some of these functions to contribute to the development of AD (typified by ‘forgetfulness and difficult‐thinking’) will be discussed.

